# A Pathogenic Missense Variant in *NFKB1* Causes Common Variable Immunodeficiency Due to Detrimental Protein Damage

**DOI:** 10.3389/fimmu.2021.621503

**Published:** 2021-04-27

**Authors:** Manfred Fliegauf, Renate Krüger, Sophie Steiner, Leif Gunnar Hanitsch, Sarah Büchel, Volker Wahn, Horst von Bernuth, Bodo Grimbacher

**Affiliations:** ^1^ Institute for Immunodeficiency, Center for Chronic Immunodeficiency (CCI), Medical Center - University of Freiburg, Faculty of Medicine, University of Freiburg, Freiburg, Germany; ^2^ CIBSS – Centre for Integrative Biological Signalling Studies, Albert-Ludwigs University, Freiburg, Germany; ^3^ Department of Pediatric Respiratory Medicine, Immunology and Critical Care Medicine, Charité – Universitätsmedizin Berlin, Berlin, Germany; ^4^ Department of Medical Immunology, Charité – Universitätsmedizin Berlin, Berlin, Germany; ^5^ Department of Immunology, Labor Berlin Charité - Vivantes GmbH, Berlin, Germany; ^6^ Berlin Center for Regenerative Therapies (BCRT), Charité – Universitätsmedizin Berlin, Berlin, Germany; ^7^ DZIF – German Center for Infection Research, Satellite Center Freiburg, Freiburg, Germany; ^8^ RESIST – Cluster of Excellence 2155 to Hanover Medical School, Satellite Center Freiburg, Freiburg, Germany

**Keywords:** NFKB1, nuclear factor kappa B subunit 1, NF-kappa B, NF-κB, CVID, Common Variable Immunodeficiency, hypogammaglobulinemia

## Abstract

In common variable immunodeficiency (CVID), heterozygous damaging *NFKB1* variants represent the most frequent monogenic cause. *NFKB1* encodes the precursor p105, which undergoes proteasomal processing to generate the mature NF-κB transcription factor subunit p50. The majority of *NFKB1* sequence changes comprises missense variants of uncertain significance (VUS), each requiring functional evaluation to assess causality, particularly in families with multiple affected members presenting with different phenotypes. In four affected members of a German family, all diagnosed with CVID, we identified a previously uncharacterized heterozygous *NFKB1* missense variant (c.1049A>G; p.Tyr350Cys). The clinical phenotypes varied markedly regarding onset, frequency and severity of infections. Consistent immunologic findings were hypogammaglobulinemia with normal specific antibody response to protein- and polysaccharide-based vaccinations, reduced switched memory B cells and decreased lymphocyte proliferation upon stimulation with the B cell mitogen SAC. To assess the pathogenicity of the *NFKB1* missense variant, we employed immunophenotyping and functional analyses in a routine *in vitro* cell culture model. Following site-directed mutagenesis to introduce the variant into overexpression vectors encoding EGFP-fused p105 or p50, we analyzed transiently transfected HEK293T cells by confocal imaging and Western blotting. The cytoplasmic p105-Tyr350Cys precursor gained only weak expression levels indicating accelerated decay. The missense change disabled processing of the precursor to prevent the generation of mutant p50. Unlike the wildtype p50, the overexpressed mutant p50-Tyr350Cys was also not sustainable and showed a conspicuous subnuclear mislocalization with accumulation in dense aggregates instead of a homogenous distribution. Electrophoretic mobility shift assays, fluorescence-based reporter gene analyses and co-transfection experiments however demonstrated, that the DNA-binding activity of p50-Tyr350Cys and the interaction with RelA(p65), IκBα and wildtype p50 were preserved. Mutation carriers had reduced p105 and p50 levels, indicating insufficient protein amounts as the most likely primary defect. In conclusion, the missense variant c.1049A>G caused a detrimental defect, preventing the persistent expression of both, the p105-Tyr350Cys precursor and the mature p50-Tyr350Cys. The variable clinical phenotypes among affected family members sharing an identical pathogenic *NFKB1* variant support a disease mechanism provoked by a p105/p50 (haplo)insufficient condition.

## Introduction

Common variable immunodeficiency (CVID; [MIM 607594]) is a highly heterogeneous disorder with broad ranges of clinical manifestations, age of onset (with most patients being diagnosed at age 20 – 45), and genetic causes (1–5). CVID has an estimated incidence of 1:25,000 to 1:50,000 and represents the most frequent symptomatic primary immunodeficiency. Key features at initial presentation are recurrent infections (mostly bacterial and viral ear, nose and throat (ENT) and airway infections) due to hypogammaglobulinemia and decreased IgA and/or IgM, as well as inflammatory complications and poor specific antibody responses. A variety of additional non-infectious complications, including autoimmune disorders, lymphoproliferative conditions, interstitial lung disease, gastrointestinal enteropathy, allergy and oncogenic malignancies are common. The loss of B cell function in CVID is reflected by specific abnormalities in the B cell subset composition: Most affected individuals have severely reduced numbers of isotype-switched memory B cells, and an expansion of CD21(low) B cells is frequent, whereas pre-germinal center B cells are preserved (2, 4, 5). Abnormal low numbers of T cell subsets and functional T cell defects have also been associated with the pathogenesis of CVID (6).

The mechanisms leading to insufficient antibody production in CVID are poorly understood. Based on the heterogenic symptoms, CVID has been proposed to comprise a group of oligo/polygenic syndromes (7). However, familial inheritance has been described in approximately 25% of CVID cases and several disease-causing monogenetic defects have been identified (8, 9). These account for 20% to 40% of the affected subjects and include mutations in the OMIM-listed genes *ICOS* (CVID1), *TNFRSF13B* (encoding TACI; CVID2), *CD19* (CVID3), *TNFRSF13C* (encoding the BAFF receptor; CVID4), *CD20* (CVID5), *CD81* (CVID6), *CD21* (CVID7), *LRBA* (CVID8), *NFKB2* (CVID10), *IL21* (CVID11), *NFKB1* (CVID12), *IKZF1* (encoding IKAROS; CVID13) and *IRF2BP2* (CVID14). Variants in additional genes (not listed as “CVID genes” in OMIM), including *BLK, CD27, CD70, CTLA4*, *IL21R, PIK3CD*, *PIK3R1, PLCG2*, *PRKCD, RAC2, TNFSF12* and *VAV1* have also been associated with CVID (4, 8–10). Routine mutational analysis, particularly by next generation and whole exome sequencing, has recently enabled the identification of typically heterozygous *NFKB1* and *NFKB2* variants, which collectively account for the most frequent genetic cause in CVID (10–24).

*NFKB1* (nuclear factor kappa B subunit 1) and *NFKB2* encode the central components of the canonical and non-canonical NF-κB (nuclear factor of kappa light polypeptide gene enhancer in B cells; NF-kappaB) signaling pathways, respectively (25–29). Both NF-κB transcription factors (sometimes referred to as class I) are expressed as longer precursors which undergo proteolytic processing of their C-terminal parts to generate the mature DNA-binding forms (NF-κB1: p105 to p50; and NF-κB2: p100 to p52). The remaining NF-κB family members are RelA (also known as Transcription factor p65), RelB, and c-Rel (class II). Active DNA-binding dimeric complexes are composed of various combinations of the NF-κB and Rel proteins (30, 31). Since neither p50 nor p52 is equipped with a transactivation domain, homodimers of p50 or p52 and p50/p52 heterodimers act as repressors when bound to their target promoters. To assemble transcriptional activators requires p50 and p52 to heterodimerize with one of the three Rel proteins, which provide a transactivation domain to the dimer. All members of the NF-κB protein family share the N-terminal Rel-homology domain (RHD), which mediates dimerization, DNA-binding and which harbors the nuclear localization sequence (NLS).

In unstimulated cells, NF-κB dimers are sequestered in the cytoplasmic compartment by inhibitory proteins (IκB), including IκBα, IκBβ, IκBϵ, and the precursors p105 and p100 itself. Numerous stimuli signal *via* NF-κB, to promote multiple processes in both innate and adaptive immunity, including cell proliferation, differentiation, anti-apoptosis, inflammation and immune responses (25–29). Initiated by activated upstream kinases (IKKα, -β and -γ complex in the canonical pathway; NIK and IKKα in the non-canonical pathway), phosphorylation, poly-ubiquitination and subsequent proteasomal degradation of IκB is mediated. The released NF-κB dimer can translocate to the nucleus, to bind to the promoter of its target genes. Although the classical model predicts NF-κB proteins to be retained in the cytoplasm by IκB proteins, these complexes continuously shuttle between the cytoplasmic and the nuclear compartments, yet with an almost exclusively cytoplasmic steady-state distribution (32).

Most patients with damaging *NFKB1* mutations have typical CVID manifestations such as hypogammaglobulinemia, low switched memory B cells, respiratory and less frequent gastrointestinal infections (20, 22, 24). Autoimmunity, lymphoproliferation, non-infectious enteropathy, opportunistic infections and autoinflammatory features are frequent but variable. In contrast, heterozygous damaging mutations in *NFKB2* represent a distinct, early-onset entity, exceeding the usual clinical spectrum of antibody deficiency, and affecting diverse lymphocyte subpopulations (33).

Severe lesions in *NFKB1* and *NFKB2*, including truncations, internal deletions (both leading to reduced levels of p50 or p52) and frameshift mutations (predicting the expression of p50-like or p52-like proteins) have been shown to cause disease. Particularly in *NFKB1*, a huge number of missense variants has been identified (22, 24). Yet, the vast majority of these single amino acid substitutions has not been functionally characterized and thus their pathogenic relevance remains obscure. Relying on genetic testing to establish a solid diagnosis, variants of uncertain significance (VUS) are disappointing for patients and treating physicians. Although *in silico* tools provide valuable pathogenicity predictions for any missense change, experimental confirmation is usually inevitable. Due to the considerable effort required, missense variants have only sporadically been analyzed yet. Three *NFKB1* missense mutants (p.Ile87Ser; p.Val98Asp; p.Ile281Met) have all been shown to cause reduced p50 levels in patient-derived cells (22). Others caused a delay of p50 nuclear translocation (p.His67Arg) or p105 precursor instability (p.Ile553Met) (15). Recently, p.Arg157Pro was shown to cause reduced expression of p105 (and thus also p50) in PBMCs whereas the EGFP-fused mutant p50 had aberrant nuclear localization in transfected cells (34). Two missense variants (p.Arg57Cys and p.Ile87Ser) showed altered reporter gene activation (24).

Here, we show that a *NFKB1* missense variant (c.1049A>C) predicting a single amino acid change (p.Tyr350Cys) in close proximity to the nuclear localization sequence (NLS), leads to detrimental damage and thus to insufficiency of both, p105 and p50 in patient-derived cells. Analyses of transfected HEK293T cells showed, that the mutant p105 precursor underwent accelerated decay instead of being further processed, whereas forced expression of mutant p50 caused mislocalization within the nucleus, although nuclear translocation, DNA-binding, and protein-protein-interactions were preserved. This convenient and reliable *in vitro* procedure enables the analysis of NF-κB defects and is based on the forced expression of both, the mutant p105 precursor and the mutant mature p50.

## Study Subjects

**Figure 1:**
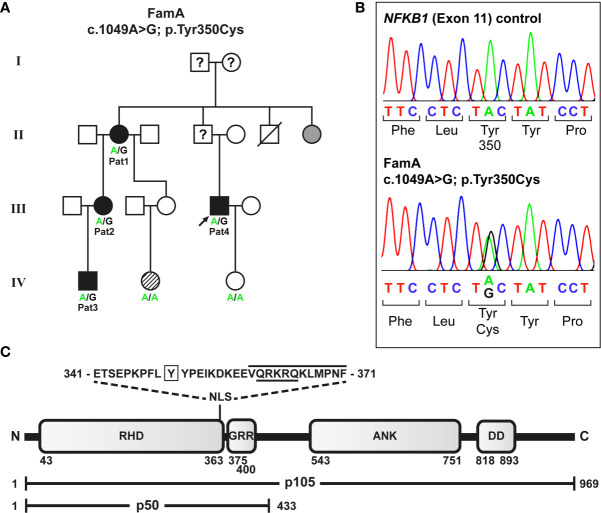
Segregation of a heterozygous *NFKB1* missense variant with variable disease phenotypes in a non-consanguineous German CVID family. **(A)** The missense variant was identified in patient 4 (index; arrow) by targeted next generation sequencing. Family members tested by Sanger sequencing are indicated. Circles, female; squares, male; filled symbols, affected individual, open symbol, healthy member; grey symbol, symptomatic but not tested; hatched symbol, unclear etiology; slash, deceased individual; question mark, unknown status. **(B)** Sequencing chromatograms confirming the heterozygous missense variant in an affected individual. **(C)** The p.Tyr350Cys amino acid change maps close to the NF-κB1 nuclear localization sequence. Protein domains of the p105 precursor (long horizontal bar) comprise the N-terminal Rel-homology domain (RHD), the nuclear localization sequence (NLS), the central glycine-rich region (GRR), and the C-terminal Ankyrin-repeat domain (ANK) and the death domain (DD). The sequence of the predicted NLS (cNLS Mapper), comprising a bi- (aa341-367; dotted lines) and a monopartite (aa360-371; black line) NLS, and the amino acid position (Y350) affected by the missense change (p.Tyr350Cys) are indicated. The NLS listed in UniProt is underlined. In the mature p50 form (short horizontal bar), which originates from limited proteasomal processing of the C-terminal half of p105, the NLS is exposed and can promote nuclear import. Amino acid positions are indicated by numbers.

Four relatives from a German non-consanguineous family (FamA) were affected (**Figure 1A**). CVID in the four individuals was diagnosed according to ESID working definitions for clinical diagnosis of inborn errors of immunity (IEI) based on a) recurrent infections, b) decreased IgG and IgA (patient 1 developed decreased IgA later in life), and c) decreased switched memory B cells.

### Patient 1 (Female)

Patient 1 developed type 2 diabetes mellitus, hypothyroidism, and restless leg syndrome in adulthood. She has had chronic gonarthritis with severe local inflammation; surgery was performed without marked improvement. At the age of 60 years, she started to suffer from recurrent bacterial airway infections leading to pneumonia twice. She reported allergic reactions to several antibiotics. Hypogammaglobulinemia was first documented at the age of 69 years. Later in life, decreased IgA levels were also observed (**Table 1**). Prior to IgG-substitution, the patient was regularly vaccinated against Tetanus and Pneumococcus (Pneumovax^®^), last time more than 3 years prior to determination of specific antibodies, which were within normal range (**Table 1**). Lymphocyte proliferation was normal upon stimulation with three mitogens (PHA, anti-CD3, PWM) and three recall antigens (candida, diphtheria, tetanus), whereas proliferation upon the predominant B cell mitogen SAC was markedly reduced (**Table 2**). B cell analyses showed a minor decrease of switched memory B cells (**Table 2**). Full and differential blood count showed normal values. IgG substitution (4-weekly IVIG) was initiated at the age of 70 years with marked reduction of infections since.

**Table 1:** Immunoglobulin levels prior to IgG substitution and lymphocyte subsets.

Patient ID	1		2	3	4
Age (blood sample)	69	75	38	4	20
**Serum immunoglobulins (g/l)**					
IgG	**5.67 ↓** (7.0-16)	(9.45*)	**4.0 ↓** (7.0-16)	**3.08 ↓** (5.04-14.64)	**2.5 ↓** (7.0-16)
IgA	0.78 (0.7–4.0)	**0.57 ↓**	0.42 **↓** (0.7 – 4.0)	0.14 **↓** (0.27-1.95)	**< 0.3** (0.7 – 4.0)
IgM	0.76 (0.4 – 2.3)	0.42	0.64 (0.4 – 2.3)	0.37 (0.24-2.1)	**< 0.27 ↓** (0.4 – 2.3)
IgG1	3.21 (2.8 – 8.0)	n.d.	2.49 (2.8 – 8.0)	2.115 (2.20-7.20)	n.d.
IgG2	2.15 (1.15-5.7)	n.d.	1.17 (1.15-5.7)	0.693 0.50-1.80	n.d.
IgG3	0.628 (0.24–1.25)	n.d.	0.285 (0.24–1.25)	0.478 0.14-0.91	n.d.
IgG4	0.227 (0.052–1.25)	n.d.	<0.001 (0.052–1.25)	0.034 (0.00- 0.41)	n.d.
**Specific antibodies**					
Tetanus-Toxoid (IU/ml)	1.28 (> 0.1)	n.d.	1.11 (> 0.1)	0.21 (> 0.1)	n.d.
Pneumococcal-IgG2 (mg/l)	39.11 (4.7– 89.4)	n.d.	5.09 (4.7– 89.4)	1.93 (0.50-87.0)	n.d.
Pneumococcal-IgG total (mg/l)	85.83 (10-191.2)	n.d.	16.69 (10-191.2)	7.64 (0.90-29.2)	n.d.
Age (blood sample)	69	75	43	6	42
**Lymphocyte subsets (cells/µl)**					
CD3	n.d.	0.6 (0.9-2.2)	1.37 (0.9-2.2)	1.43 (0.7-4.2)	1.55 (0.9-2.2)
CD4	n.d.	0.3 (0.5-1.2)	0.85 (0.5-1.2)	0.70 (0.3-2.0)	0.53 (0.5-1.2)
CD8	n.d.	0.28 (0.30-0.80)	0.49 (0.3-0.8)	0.57 (0.3-1.8)	0.98 (0.3-0.8)
NK	n.d.		0.12 (0.1-0.4)	0.4 (0.09-0.90)	0.15 (0.1-0.4)
B cells (CD19+)	n.d.	0.10 (0.1-0.4)	0.40 (0.1-0.4)	0.36 (0.2-1.6)	**0.04 ↓** (0.1-0.4)
**B cell subsets (%)**					
Naïve (CD19+CD27-/IGD+	67.1 (42.6-82.3)	68	**40.1 ↓** (42.6-82.3)	**62.0 ↓** (67.8-89.0)	76.4 (42.6-82.3)
Marginal zone (CD19+/CD27+/IgD+/IgM+)	12.9 (7.4-32.5)	8.2	**49.08 ↑** (7.4-32.5)	**18.8 ↑** (5.00-16.20)	4.4 (7.4-32.5)
IgM only (CD19+/CD27+/IgD-/IgM+)	4.6	2.1	0.86	0.6	4.0
Switched memory (CD19+/CD27+/IgD-/IgM-)	**5.1 ↓** (6.5-29.1)	**3.9 ↓**	**2.24 ↓** (6.5-29.1)	**3.0 ↓** (4.0-14.00)	**0.3 ↓** (6.5-29.1)
Transitional (CD18+/CD38++/CD24+)	**4.2 ↑** (0.60-3.40)	2.5	1.03 (0.60-3.40)	2.1 (0.6-3.4)	**7.9 ↑** (0.6-3.4)
Activated (CD19+/CD21low/CD38low)	5.9 (0.9-7.6)	9.7	12.51 ↑ (0.9-7.6)	**12.7 ↑** (0.90-7.60)	1.6 (0.9-7.6)
Switched plasmablasts (CD19+/CD27++/CD38++)	**0.3 ↓** (0.4-3.6)	**0.2 ↓**	1.21 (0.4-3.6)	0.5 (0.4-3.6)	0.5 (0.4-3.6)
**T cell subsets (%)**					
α/β TCR+ T cells	n.d.	n.d.	98	90	96
γ/δ TCR+ T cells	n.d.	n.d.	2	10	4
**T cell subsets (% of CD4)**					
Naïve (CD45RA+CCR7+)	n.d.	n.d.	37.98 (17.46-60.24)	59.9	**16.03 ↓** (17.46-60.24)
TEMRA (CD45RA+CCR7-)	n.d.	n.d.	**0.19 ↓** (2.74-15.54)	1.35	**0.06 ↓** (2.74-15.54)
Central memory (CD45RA-CCR7+)	n.d.	n.d.	**47.29 ↑** (16.4-33.41)	29.18	**66.84 ↑** (16.4-33.41)
Effector memory (CD45RA-CCR7-)	n.d.	n.d.	**14.54 ↓** (17.38-40.38)	9.75	**17.07 ↓** (17.38-40.38)

Numbers in brackets indicate normal reference ranges; bold numbers and arrows indicate values higher than (↑) or lower than (↓) the reference ranges; n.d., not determined; * under IgG substitution.

**Table 2:** Lymphocyte proliferative response upon stimulation with mitogens.

Patient ID	1	2	3	4
Age (blood sample)	74	42	6	44
**Mitogen (normal SI)**				
SAC (>10)	**1.6**	**4.4**	**8.5**	**3.4**
(control)	**(5.7)**	(16.5)	(11.6)	(16.5)
PWM (>24)	24	81.9	67.8	75.8
(control)	(74.8)	(191.9)	(204.1)	(191.9)
PHA (>50)	242.7	681.9	478.9	462.2
(control)	(385.2)	(964.5)	(780.7)	(964.5)
Anti-CD3 (>30)	76.0	179.8	167.0	258.0
(control)	(148.1)	(119.3)	(164.2)	(119.3)
Il-2 (>30)	**5.3**	40.8	71.2	41.2
(control)	(31.9)	(87.1)	(71.5)	(87.1)

Results are shown as stimulation indices (SI) with results from one healthy control (in parentheses) for each analysis. Bold numbers indicate values lower than the reference values. Il-2, Interleukin-2; PHA, Phytohemagglutinin; PWM, Pokeweed mitogen; SAC, S. aureus Cowan strain; SI, stimulation index.

### Patient 2 (Female)

During childhood, patient 2 (daughter of patient 1; **Figure 1A**) suffered frequently from viral and bacterial infections (in particular tonsillitis). She was sick up to 240 days per year. After tonsillectomy at the age of seven years, infections occurred less frequently. In adulthood, she suffered from recurrent upper respiratory tract infections and bronchitis (twice per year), and sinusitis (once – twice per year). Occasionally, she had labial herpes. Asthma, triggered by infections, was diagnosed at the age of 13. She was sensitized against cat allergen (specific IgE). As an adult, she suffered from a severe anaphylactic reaction to macadamia nuts with detection of high specific IgE antibodies. She had minimal vitiligo and a mild splenic enlargement. Recurrent migraine episodes started at the age of 25 years. She complained of occasional stomach aches, nausea, vomiting and diarrhea. Endoscopy was recommended but had not been performed. Following the diagnosis of her mother, decreased IgG, IgA, and IgG1 were detected at the age of 38 years (**Table 1**). Specific antibodies (tetanus-toxoid and pneumococcal antigens) were low normal, vaccination with Pneumovax^®^ (first pneumococcal vaccination) resulted in an >3-fold increase of specific antibodies. She had reduced switched memory B cell numbers. Lymphocyte proliferation was normal upon stimulation with 4 mitogens (PHA, IL-2, anti-CD3, PWM) and 3 recall antigens (candida, diphtheria, tetanus), whereas proliferation upon the predominant B cell mitogen SAC was markedly reduced (**Table 2**). Full and differential blood counts were normal. IgG substitution therapy (weekly SCIG) was initiated at the age of 40 years with very good clinical response.

### Patient 3 (Male)

Patient 3 (son of patient 2; **Figure 1A**) had suffered from very frequent viral respiratory tract infections and febrile episodes since the age of 2 years, he had no bacterial infections. Hypogammaglobulinemia was first diagnosed at the age of 2 years, following the diagnosis of his grandmother (**Table 1**). Specific antibodies (tetanus-toxoid and pneumococcal antigens) were within the lower normal range, vaccination with Pneumovax^®^ (first pneumococcal vaccination) resulted in a normal (>3-fold) increase of specific antibodies. B cell and T cell subsets are shown in **Table 1**. Lymphocyte proliferation was normal upon stimulation with the mitogens PHA, IL-2, anti-CD3, and PWM, and three recall antigens (candida, diphtheria, tetanus), whereas proliferation to the predominant B cell mitogen SAC was markedly reduced (**Table 2**). He was sensitized against birch and grass pollen (specific IgE). IgG-substitution (weekly SCIG) was initiated at age of 4 years with very few mild upper respiratory tract infections thereafter.

### Patient 4 (Male)

Patient 4 (index patient; cousin of patient 2 and nephew of patient 1; **Figure 1A**) had suffered from recurrent viral and bacterial airway and ENT infections since early childhood. As an adult, he had 5 to 10 episodes of severe pneumonia (necessitating 4 times intravenous antibiotic treatment). At the age of 33 years (under replacement with IgG) he was diagnosed with paratyphus with pleural empyema. Idiopathic thrombocytopenia (ITP) was diagnosed at the age of 11 years. He was treated as an in-patient for 6 months with no response to high dose and prolonged therapy with glucocorticoids. Platelet counts increased spontaneously to normal levels in puberty and remained within low normal levels since then. Splenomegaly was documented at the age of 20 years. Despite IgG substitution with IgG trough levels around 8 g/L, he has still suffered from recurrent lower airway infections (5 to 6 times per year). Bronchiectases were diagnosed in his 30ies. Five molar teeth were extracted after root infections. Hypogammaglobulinemia and CD4 lymphopenia (0.36/nl) was documented at the age of 20 years (**Table 1**). B cell and T cell subsets at age 42 are shown in **Table 1**. Lymphocyte proliferation was normal upon stimulation with 4 mitogens (PHA, IL-2, anti-CD3, PWM) and 3 recall antigens (candida, diphtheria, tetanus), whereas proliferation upon the B cell mitogen SAC was reduced (**Table 2**). IgG substitution (weekly SCIG) was started at the age of 27 years with persisting but fewer infections thereafter. An increase of IgG-dosing with trough levels > 10g/l was recommended.

## Materials and Methods

### Mutational Analysis

Genetic analysis was performed by Targeted Next Generation Sequencing as previously described (12, 33). Briefly, genomic DNA was purified from Peripheral blood mononuclear cells (PBMCs) using QIAamp kits (Qiagen, Hilden, Germany) followed by Halo-Plex target enrichment (Agilent, Waldbronn, Germany) and amplification of the captured target libraries. Samples were subjected to multiplex sequencing on an IlluminaMiSeq system (Illumina, Eindhoven, The Netherlands) using Illumina v2 reagent kits. The sequence variant was confirmed in family members by Sanger sequencing. The frequency of the identified variant was reviewed in public databases SNPbase (www.ncbi.nlm.nih.gov/snp), ENSEMBL, (www.ensembl.org) and Genome aggregation database gnomAD (https://gnomad.broadinstitute.org/).

### Cell Culture and Transfection

HEK293T cells were grown in DMEM/10%FCS/1% penicillin/streptomycin in standard cell culture flasks. Cells were plated onto collagen-A-coated 48-well culture plates or onto glass cover slips (placed in 24-well plates) 3-6 hours prior to transfection with the indicated cDNA expression vector constructs (typically 150-300 ng plasmid DNA per well for single vector transfections) using X-tremeGENE HP reagent (Roche, Mannheim, Germany). To ensure equal transfection efficiencies, conditions were kept constant using identical reagents in each experiment. In general, transfections with single vectors were largely in-sensitive to variations, whereas co-transfections were dependent on appropriately titrated vector ratios. DNA amounts were equalized with a non-relevant plasmid DNA when appropriate. Cells were harvested or analyzed within 40-48 hours after transfection, if not indicated otherwise. Detailed protocols are available from the authors upon request.

### Generation of cDNA Expression Vectors and Site-Directed Mutagenesis

The vector constructs encoding the N-terminally EGFP-tagged wildtype p105 and p50 and the truncated p105Δex8 and p50Δex8 subcloned into pEGFP-C1 (Clontech, Takara, Saint-Germain-en-Laye, France) have previously been described (12). Mutations were introduced by site-directed mutagenesis using overlap-extension PCR. The cDNAs encoding RelA(p65) and IκBα, were amplified by RT-PCR from an in-house healthy volunteer RNA source. Non-fused versions were generated by re-cloning of the cDNAs (including a stop codon) into pEGFP-N1 and mScarlet fusion constructs were generated by re-cloning into pmScarlet-C1 (35). More detailed information and all vector constructs are available from the authors upon request.

### Western Blot Analysis of Transfected Cells

Cells were washed with phosphate buffered saline (PBS) and lysed on ice in RIPA buffer (50 mM Tris pH 8, 1% Igepal, 0.5% sodium-deoxycholate, 150 mM NaCl, 1 mM ethylenediaminetetraacetic acid (EDTA), 0.1% SDS, 1% protease inhibitor cocktail). Supernatants were separated on discontinuous 4%/9% Bis-Tris polyacrylamide gels and transferred onto PVDF membranes (Merck/Millipore, Darmstadt, Germany) according to standard methods. p105 and p50 were detected with a rabbit antibody raised against the N-terminus of NF-κB1 (#3035; Cell Signaling, Frankfurt, Germany) or against residues surrounding Ile415 of mouse NF-κB1 (#13586, Cell Signaling). Mouse-anti-beta actin antibodies (A2228, Sigma-Aldrich/Merck or #3700, Cell Signaling) were used as controls. Signals were detected with IRDye-coupled (LI-COR Biosciences, Bad Homburg, Germany) or DyLight-coupled (Cell Signaling) anti-rabbit and anti-mouse secondary antibodies with an Odyssey CLx infrared scanner (LI-COR).

### Electrophoretic Mobility Shift Assay

Nuclear protein extracts were prepared from cells transfected on 48-well plates following a previously described procedure with minor modifications (36). Binding reactions were carried out at room temperature with DY681-labelled annealed oligos (forward 5`– AGT TGA GGG GAC TTT CCC AGG C – 3` and reverse 5`- GCC TGG GAA AGT CCC CTC AAC T -3`) in a total volume of 10µl in 1x binding buffer (10mM Tris pH 7.4; 1mM EDTA; 100mM KCl; 0.25mM DTT; 0.25% Tween-20; 5% glycerol; 0.01% BSA; 100ng/µl poly-dI:dC). Unlabeled and unlabeled/mutant probes were used as controls in pre-experiments to confirm specific binding and compensation, respectively (not shown). Samples were separated on 6% polyacrylamide mini gels with 1xTAE running buffer and visualized with an Odyssey CLx infrared scanner (LI-COR).

### Fluorescence-Based Reporter Assay

The transcriptional activating function of wildtype and mutant p105/p50 was assessed with a fluorescence-based promoter reporter assay in transiently transfected HEK293T cells (in duplicates or triplicates) on 48-well plates. A synthetic promoter reporter vector construct was generated, in which the red fluorescent protein tdTomato (Takara/Clontech, Saint-Germain-en-Laye, France) is under the control of a NF-κB responsive promoter (composed of five NF-κB consensus binding sites [TGGGGACTTTCCAC]_5_, fused to the CMV minimal promoter). In pre-experiments (not shown), a vector in which the tdTomato sequence was fused to the CMV minimal promoter (lacking specific transcription factor binding sites) was used as a background control, while a vector with the full-length CMV promoter driving tdTomato expression was used as positive control. Expression vector constructs for wildtype or mutant p105 or p50 (typically 300 ng per well if not indicated otherwise) were transfected together with the reporter (100 ng per well) and, to provide a transactivation domain, a wildtype form of non-fused RelA (5 ng per well) into HEK293T cells. DNA amounts were titrated in pre-experiments (not shown) to maximize the effects and may be variable as indicated. Fluorescence intensities were examined in live cells as an indicator of reporter activity and were visualized by automated epi-fluorescence microscopy of each well using a FluoroSpot Analyzer (CTL Immunospot, Bonn, Germany). Plates were usually recorded 48 and/or 72 hours after transfection with variable exposure times and magnifications, to aim either at best visibility or accurate quantification. The mean fluorescence intensity in the microscopic images of each well was quantified using the freely available ImageJ software (37). Fold-changes of fluorescence signals over background were calculated with the “reporter only” control defined as 1. Detailed guidelines and plasmid constructs can be obtained from the authors upon request.

### Fluorescence Microscopy

For confocal imaging, transfected cells grown on collagenized glass cover slips (placed in 24-well plates) were rinsed with PBS, fixed with 4% paraformformaldehyde for 15 min at RT and nuclei were stained with Hoechst 33342 (Sigma-Aldrich/Merck). Cover slips were mounted onto glass slides using fluorescence mounting medium (Dako/Agilent, Frankfurt, Germany). A Zeiss laser scanning microscope LSM710 equipped with a 63x/1.40N.A. oil immersion objective (Carl Zeiss, Jena, Germany) was used for confocal imaging. Images were processed with the Zeiss ZEN-black software. In all other experiments, transfected live cells were directly monitored in standard cell culture vessels without further treatment during the transfection process (e.g. after over-night incubation and before cells were collected for the experiment) using a conventional epi-fluorescence microscope.

### Analysis of Patient-Derived Blood Cells and Immunophenotyping

PBMCs were isolated from fresh blood samples by ficoll density gradient centrifugation and cultured in RPMI 1640 Medium supplemented with 10% fetal bovine serum and 1% penicillin/streptomycin. Cells were stimulated with 50 ng/ml phorbol 12-myristate 13-acetate (PMA) and 1 mg/ml ionomycin (final concentrations; both from Sigma-Aldrich/Merck) and harvested using lysis buffer (50 mM Tris-HCl pH8, 1% NP-40, 0.5% sodium-deoxycholate, 150 mM NaCl, 1 mM ethylenediaminetetraacetic acid (EDTA), 0.1% SDS, 1% protease inhibitor cocktail) at the indicated time-points. Cell lysates were subjected to standard 5%/10% Lämmli SDS page followed by Western blotting. Antibodies were: rabbit polyclonal anti-p105/p50 and rabbit monoclonal anti-phosphorylated p105 (#3035 and #4806, both from Cell Signaling). Rabbit monoclonal anti-ß-actin (622102, Biolegend, Koblenz, Germany) and monoclonal mouse anti-GAPDH (#97166, Cell Signaling) were used as controls. HRP-coupled secondary antibodies were from Cell Signaling (#7074; #7076). Signals were detected using enhanced chemiluminescence (Thermo Fisher Scientific, Darmstadt, Germany). Immunophenotyping of PBMCs was performed by staining for various cell surface markers as indicated. Immortalized B lymphocytes were generated by treatment of isolated PBMCs with Epstein-Barr-Virus (EBV) containing B95-8 cell culture supernatant.

## Results

Although all four affected members of a non-consanguineous German family showed differences in the frequency and severity of infections, they had hypogammaglobulinemia, reduced switched memory B cells and a reduced lymphocyte proliferation upon stimulation with a predominant B cell mitogen (SAC) in common. Furthermore, specific antibodies against protein and polysaccharide antigens (tetanus-toxoid, pneumococcus) were detected within (lower) normal ranges in patient 1, 2 and 3 (not tested prior to IgG-substitution in patient 4).

### Identification of a Pathogenic *NFKB1* Missense Variant in a German CVID Family

To identify the genetic origin of the disease-phenotype in the affected family (FamA) with suspected familial CVID (**Figure 1A**) we analyzed the index patient (Patient 4) by targeted next generation sequencing. A heterozygous missense variant c.1049A>G (NM_003998.4(NFKB1_v001):c.1049A>G) was identified in exon 11 of *NFKB1* (GRCh37(hg19), Chr4, NC_000004.12:g.102584803A>G) predicting the substitution of tyrosine at amino acid position 350 by cysteine (NM_003998.4(NFKB1_i001):p.(Tyr350Cys)). The single base-pair change was subsequently identified in all three additionally tested affected family members by Sanger sequencing (**Figure 1B**), but not in two unaffected members. We hypothesized that the sequence variant might lead to a functional defect of p105 and/or p50 and impaired NF-κB1 signaling as the primary cause of the family’s condition. Concordantly, the change of the conserved amino acid Tyr350 (data not shown) gained high scores using *in silico* prediction of deleterious effects (PolyPhen2 (http://genetics.bwh.harvard.edu/pph2): probably damaging score 1.0; MutationTaster (www.mutationtaster.org): disease-causing, score 194; SIFT (https://sift.bii.a-star.edu.sg): deleterious, score 0.00); and was neither listed in SNPbase nor in gnomAD or ENSEMBL.

### The p.Tyr350Cys Missense Change Causes Decay of Cytoplasmic p105 and Nuclear p50

**Figure 2:**
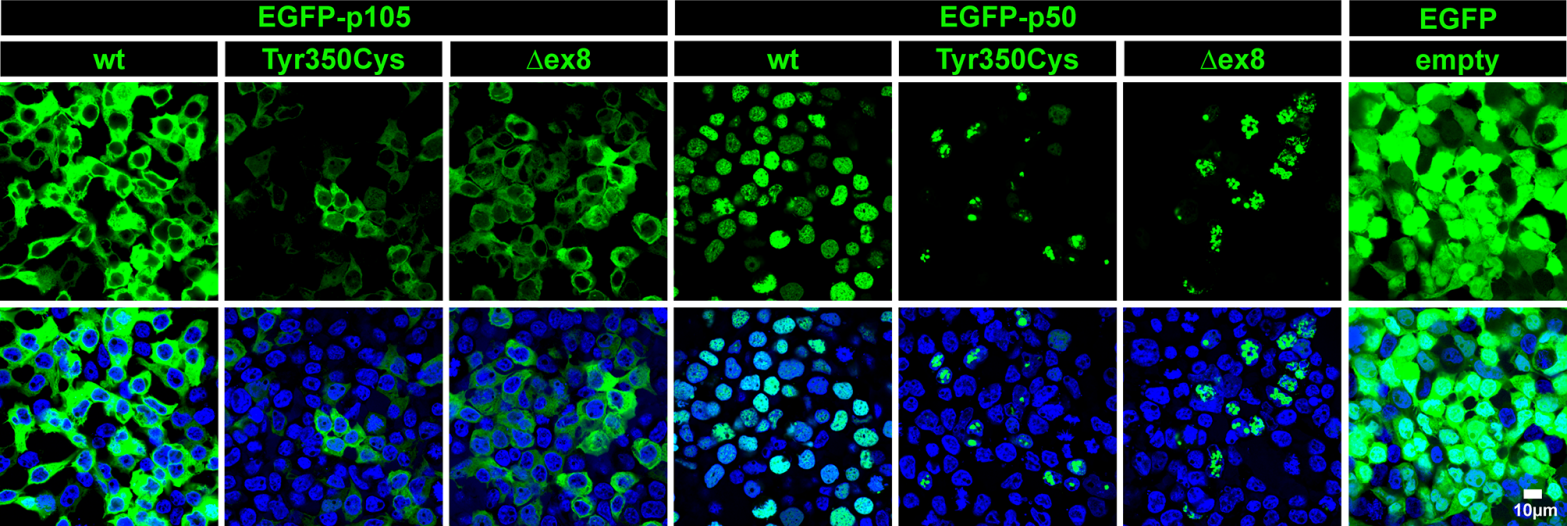
The single amino acid change p.Tyr350Cys causes limited expression of p105 and subnuclear mislocalization of p50. In transiently transfected HEK293T cells, fluorescence imaging of EGFP-tagged proteins (green) confirms normal cytoplasmic localization but limited expression of the p105-Tyr350Cys missense variant, similar to the p105Δex8 internal deletion mutant (representing a pathogenic in-frame deletion of the cDNA sequences corresponding to exon 8 of *NFKB1*). The p50-Tyr350Cys shows an aberrant subnuclear localization pattern and accumulates in high-intense spot-like structures, indistinguishable from p50Δex8, whereas wildtype p50 shows a uniform distribution within the nuclei (blue). Detector settings within the p105 and p50 panels were kept constant to demonstrate the differences in fluorescence intensities. No specific sub-cellular localization is obtained with the empty vector control. Identical results were observed at different time points (24-72 hours) using various DNA amounts. Representative examples (300 ng DNA each; 48 hours after transfection) are shown. Scale bar: 10µm. High-magnification images are shown in **Supplementary Figure 1**.

The predicted missense change (p.Tyr350Cys) affects an amino acid residue at the C-terminal end of the Rel-homology domain (RHD) in close proximity to the nuclear localization sequence (NLS; 361-QRKRQ-365; as annotated in UniProt; www.uniprot.org). An *in silico* NLS analysis tool (cNLS mapper; nls-mapper.iab.keio.ac.jp), maps a strong mono-partite NLS to amino acids 360-371, and indicates that Tyr350 is embedded in a weaker bi-partite NLS, comprising amino acids 341-371 (**Figure 1C**). Since the NLS is assumed to be inactive in the cytoplasmic p105 precursor and only exposed upon p50 processing, we hypothesized the pathogenic variant to leave p105 unaffected but to inhibit nuclear translocation of p50. We therefore generated N-terminally EGFP-tagged fusion constructs of p105-Tyr350Cys and p50-Tyr350Cys and analyzed the subcellular localization in transiently transfected HEK293T cells by confocal fluorescence imaging **(** **Figure 2**). Surprisingly, while localizing to the cytoplasm, p105-Tyr350Cys showed substantially weaker fluorescence intensities compared to the wildtype p105 upon CMV-promoter-driven overexpression, indicating intensified protein degradation. A similar reduction of the EGFP signal was obtained with the mutant p105Δex8 (harboring an internal deletion resulting from in-frame skipping of exon 8), which we have previously shown to undergo rapid decay thereby causing p50 haploinsufficiency (12). In contrast, the missense mutant p50-Tyr350Cys showed an overt abnormal subnuclear staining pattern, with spot-like accumulation of the fluorescence signal, whereas overexpressed wildtype p50 had a homogeneous intra-nuclear staining (**Figure 2**; high-magnification images are shown in **Supplementary Figure 1**). Intriguingly, the mislocalized p50-Tyr350Cys - harboring only a single amino acid substitution - resembled the aberrant pattern observed with the deleterious p50Δex8 mutant. We concluded, neither the mutant p105 precursor nor the mutant p50, are maintained at high levels in transfected cells (resulting from forced CMV-promoter-driven overexpression) but instead undergo rapid cytoplasmic decay or proteotoxic aggregation within the nucleus, respectively. The subnuclear deposition of severely damaged p50 proteins upon experimental overexpression has previously been observed with other mutations (12, 24, 34). Severely damaged proteins undergoing enhanced decay, typically gain substantially lower fluorescence signals, which might further decrease over time during the experiment. Since EGFP-fused p50-Tyr350Cys was accumulating within the nucleus, nuclear translocation and the function of the NLS were obviously unaffected.

### The Missense Defect of the p105-Tyr350Cys Precursor Disables Processing to p50

**Figure 3:**
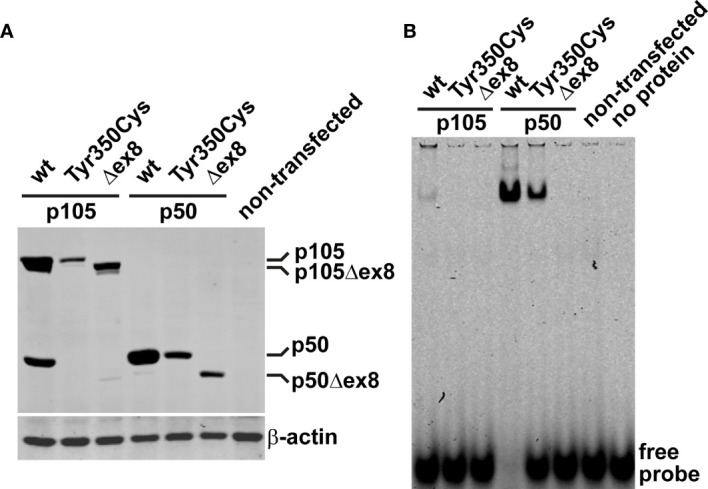
The missense variant p.Tyr350Cys causes decay of the p105 precursor but does not prevent p50 nuclear translocation and DNA-binding. **(A)** HEK293T cells were transiently transfected with EGFP-fusion constructs of wildtype or variant p105 or p50 (300 ng each per well on 48-well plates). The variant cDNAs carry either the Tyr350Cys missense change or the internal deletion Δex8, as indicated. Whole cell lysates were prepared 40 hours after transfection and analyzed by Western blotting for expression and processing of EGFP-p105 and expression of EGFP-p50, respectively, using an antibody directed against both p105 and p50. In transfected cells, a proportion of the ectopic wildtype p105 is converted to p50 by endogenous mechanisms. In contrast, p105-Tyr350Cys only gains moderate expression levels and processing to p50-Tyr350Cys is almost undetectable, resembling the internal deletion variant p105Δex8. Only limited expression of either of the variant p50 forms is obtained, unlike their wildtype counterpart. An anti-β-actin antibody was used to confirm equal loading. A representative example of at least eight experiments is shown. Similar results were obtained using various DNA amounts (100-300ng per well). **Supplementary Figure 2** shows an analogous experiment using non-fused constructs. **(B)** Nuclear proteins were isolated from transiently transfected HEK293T cells (300ng of the fusion constructs per well on 48-well plates; 48 hours) and NF-κB-DNA-binding was determined by EMSA. DNA-binding activity originating from processing of the p105 precursor to p50 is only detectable upon ectopic expression of wildtype p105 but not upon transfection of the p105-Tyr350Cys missense variant or the p105Δex8 variant. Transfection of p50-Tyr350Cys produces substantially less DNA-binding activity compared to wildtype p50, which is probably due to lower expression. In-frame skipping of exon 8, deletes a part of the Rel-homology domain and abrogates DNA-binding in the p50Δex8 variant. Representative results of at least four independent experiments are shown.

*In vivo*, limited proteolysis of the longer precursor p105 generates the mature p50. In transfected cells, a robust fraction of the ectopic wildtype p105 (regardless of whether fused to EGFP or not) is converted to p50 (or EGFP-p50, respectively) by endogenous processes (12). We therefore analyzed next, whether the mutant p105-Tyr350Cys precursor gives rise to a mature but mutant p50-Tyr350Cys. Western blotting of crude extracts of cells transfected with EGFP-fusion constructs showed the missense mutant p105-Tyr350Cys only gaining limited expression levels (**Figure 3A**; an analogous experiment using non-fused proteins is shown in **Supplementary Figure 2**), consistent with the weak signal intensities observed in our microscopic analyses. In contrast to the wildtype p105, processing of the mutant p105-Tyr350Cys precursor was almost undetectable, while the overexpressed p105Δex8 internal deletion variant produced weakly detectable amounts of p50Δex8. In addition, forced expression of the missense mutant p50-Tyr350Cys and the p50Δex8 both gained substantially lower protein levels compared to the wildtype p50. These observations suggest that the missense mutant p105-Tyr350Cys precursor might undergo rapid elimination and is only inefficiently processed to produce a mutant p50-Tyr350Cys (which also undergoes decay). Thus, as an extrapolation based on our *in vitro* observations, the heterozygous missense mutation c.1049A>G/p.Tyr350cys potentially provokes a p105/p50 insufficient condition *in vivo*.

### Nuclear Translocation and DNA-Binding Ability of the Rel-Homology Domain Remain Preserved Despite the p.Tyr350Cys Missense Change

In cells transfected with wildtype p105 (we used the EGFP-fused versions), moderate DNA-binding activity was detectable in nuclear protein extracts by EMSA (**Figure 3B**). This DNA-binding activity apparently originates from p50, generated by processing of the ectopically expressed p105, of which a small proportion translocates to the nucleus. In contrast, upon transfection of the mutant p105-Tyr350Cys, DNA-binding activity was undetectable, again indicating the mutant precursor being not or only inefficiently processed to produce a mature DNA-binding nuclear protein. Similarly, with transfection of the mutant p105Δex8, no DNA-binding-activity was detected (which here is due to an internal deletion within the Rel-homology domain, which abrogates DNA-binding). However, forced expression of the nuclear p50-Tyr350Cys demonstrates that the missense change does not prevent binding to DNA, in contrast to the DNA-binding defective p50Δex8. Therefore, although the p.Tyr350Cys missense change abolishes neither nuclear translocation, nor DNA-binding, the defect is highly damaging as indicated by decay of mutant p105 and subnuclear deposition of mutant p50.

### The Single Amino Acid Change Tyr350Cys Causes a Severe Defect of the Mature p50 Transcription Factor

**Figure 4:**
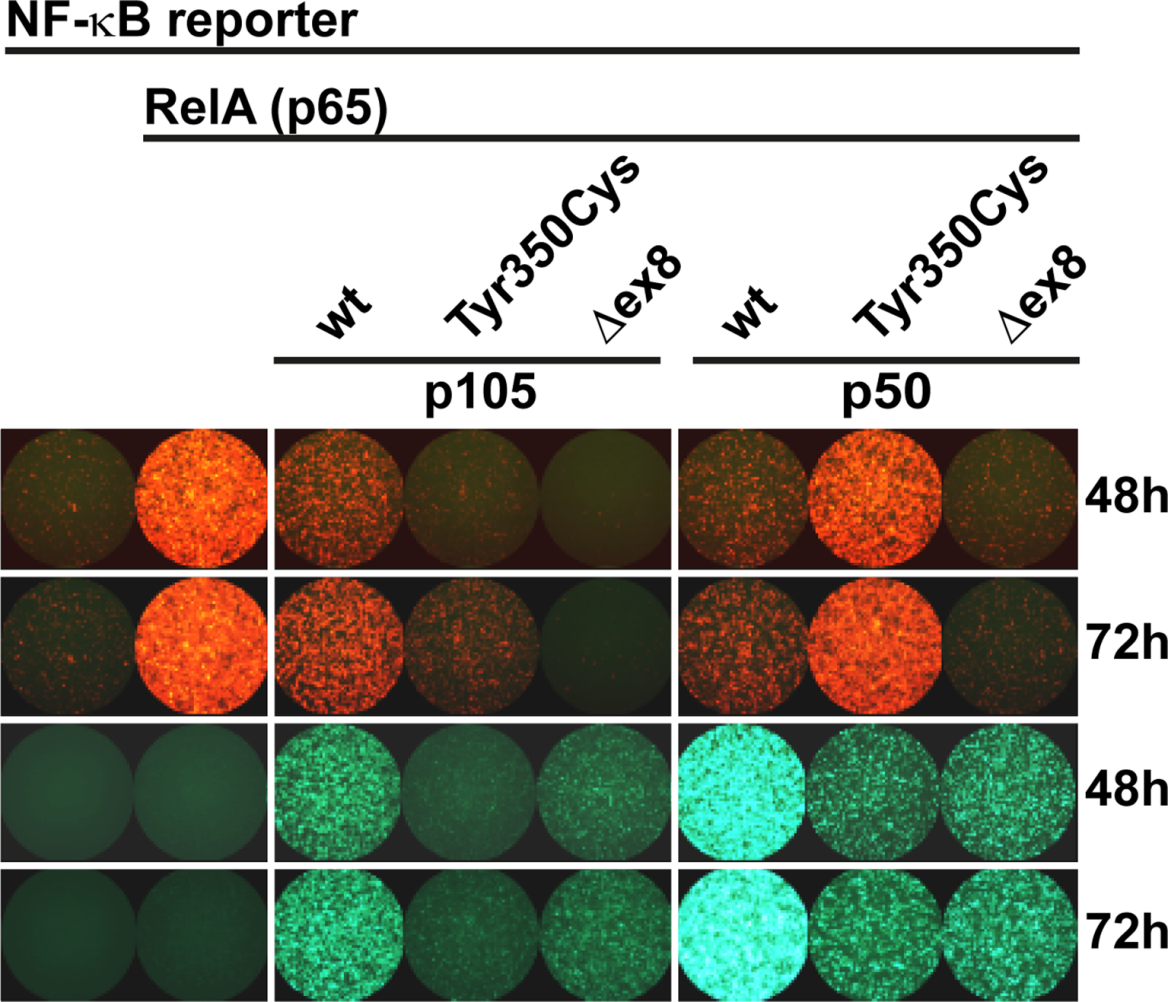
RelA interaction is preserved in p105 and p50 harboring the Tyr350Cys amino acid change. A synthetic promoter-reporter construct (100ng) harboring five NF-κB binding sites fused to the CMV minimal promoter driving a red fluorescence (tdTomato) reporter was co-transfected with a RelA(p65) vector (5ng) and an excess of EGFP-p105 and EGFP-p50 expression vectors (300ng each). Fluorescence signals were recorded at the indicated time points after transfection. (Left panel****) Reporter activity is at threshold without RelA (defined as 1-fold) whereas transfection of RelA alone allows the assembly of potent transcriptional activators and leads to strong reporter expression (12.86 ± 2.60 fold change over background at 48h; 16.13 ± 2.35 at 72h). (Middle panel****) Overexpression of wildtype p105 (3.10 ± 0.30 at 48h; 5.66 ± 1.06 at 72h) leads to moderate RelA-dependent reporter activity (which might be due to p50 processing and assembly of less potent heterodimeric p50/RelA transcriptional activators and retention of RelA by cytoplasmic p105, respectively). In contrast, p105-Tyr350Cys (1.07 ± 0.06 at 48h; 2.48 ± 0.33 at 72h) - but not p105Δex8 (0.72 ± 0.04 at 48h; 0.63 ± 0.07 at 72h) - gains only marginal reporter activity. (Right panel****) Overexpression of wildtype p50 (which can promote the assembly of homodimeric transcriptional repressors due to the lack of a transactivation domain), efficiently represses RelA-dependent reporter activation (3.10 ± 0.96 at 48h; 4.11 ± 1.03 at 72h). Transfection of p50-Tyr350Cys (gaining only residual DNA-binding activity) moderately competes with RelA-mediated reporter activation (8.20 ± 1.62 at 48h; 12.38 ± 2.49 at 72h), whereas p50Δex8 (which lacks DNA-binding activity) blocks RelA-induced reporter expression (1.61 ± 0.23 at 48h; 1.65± 0.29 at 72h). Recording the EGFP channel (lower panels****) confirms the limited expression levels of both mutant proteins, and nuclear p50 gaining higher fluorescence intensities compared to the cytoplasmic proteins, which is consistent with Western blotting and microscopic observations, respectively. Representative results of three independent experiments performed in triplicates on 48-well plates are shown. Mean fluorescence intensities were quantified with ImageJ in up to six microscopic scans of each plate at 48 and/or 72 hours after transfection and calculated as fold-change with background signals (reporter vector only defined as 1). Comparable results were obtained under modified experimental conditions (not shown).

Since the mutant p105-Tyr350Cys precursor undergoes intensified decay and nuclear p50-Tyr350Cys (which probably does not emanate from a mutant precursor *in vivo*) is displaced, we hypothesized neither of the mutant proteins to be capable of generating normal transcription factor activity. We therefore employed a reporter assay based on transient transfections of HEK293T cells, in which a synthetic NF-κB-responsive promoter drives the expression of a red fluorescent protein (**Figure 4**). The assay was adapted to override the endogenous NF-κB pathway by co-expressing an excess of wildtype or mutant p105 or p50 together with threshold amounts of RelA(p65), which provides the activation domain. Since p50 itself is not equipped with a transactivation domain, and p50:p50 homodimers act as transcriptional repressors, activation of the co-delivered reporter construct is dependent on the presence and dose-dependent availability of RelA. Whereas the highest possible reporter activation was achieved upon transfection of RelA alone (promoting RelA homodimers), reporter activity remains at baseline levels without RelA (only stimulated by endogenous NF-κB) (**Figure 4** left panel). Co-transfection of wildtype p105 together with RelA (**Figure 4** middle panel****) led to moderate activation of the reporter, consistent with partial processing to p50 and assembly of p50/RelA heterodimers while the majority of RelA molecules most likely remained engaged by cytoplasmic p105. In contrast, the mutant p105-Tyr350Cys, which undergoes accelerated decay, led only to a marginal increase of the RelA-mediated reporter activity. The difference was less prominent, when lower amounts of the p105 expression vectors were used (data not shown). The internal deletion variant p105Δex8 was unable to increase reporter activity when co-expressed with RelA.

As expected, co-expression of excess wildtype p50 led to a strong reduction of the RelA-mediated reporter activity (**Figure 4** right panel****), most likely due to the stoichiometrically preferred assembly of p50:p50 homodimeric transcriptional repressors, which block the NF-κB binding sites on the reporter. In contrast, upon co-transfection of p50-Tyr350Cys, RelA-activated reporter expression was only moderately attenuated, indicating that the limited DNA-binding activity achieved upon p50-Tyr350Cys expression was insufficient to out-compete RelA. The repressive effect was similarly neutralized, with lower relative amounts of p50-Tyr350Cys expression vector or with higher amounts of RelA (data not shown). Therefore, if a sufficient amount of nuclear protein is enforced, the p50-Tyr350Cys mutant could theoretically interfere with RelA-mediated transcription (see below). On the other hand, co-transfection of p50Δex8 almost completely blocked RelA-induced reporter expression (**Figure 4** right panel), suggesting that the loss of DNA-binding of p50Δex8 also prevents RelA from binding to the reporter. Together, these observations show that the Tyr350Cys missense mutation at the C-terminal end of the Rel-homology domain causes a harmful defect of the mature p50 (which is however, neither based on disturbed nuclear translocation, nor on DNA-binding, dimer formation, or on RelA interaction).

### The c.1049A>G/p.Tyr350Cys Missense Variant Causes p105/p50 (Haplo)Insufficiency in Heterozygous Mutation Carriers

**Figure 5:**
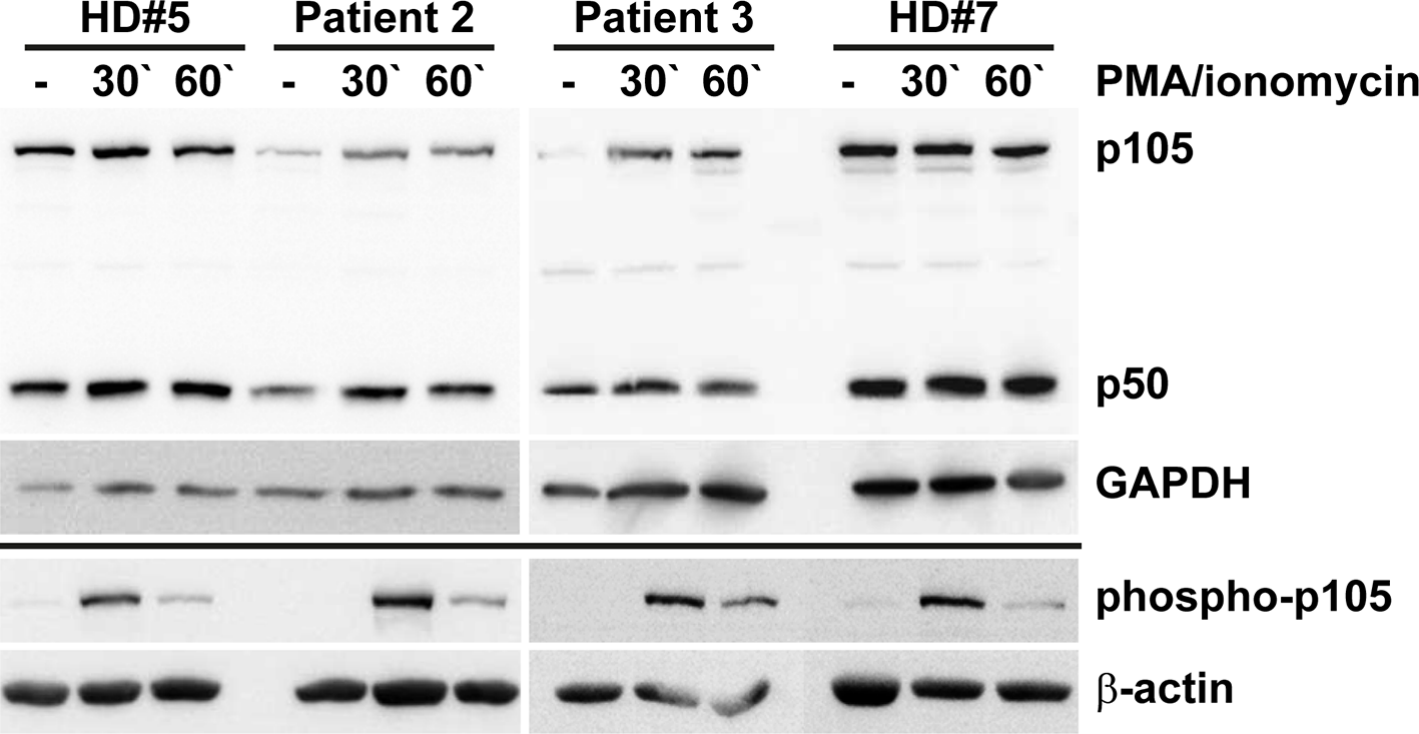
Insufficiency of p105 and p50 in heterozygous carriers of the *NFKB1* missense variant c.1049A>G/p.Tyr350Cys. PBMCs derived from two affected family members (Patients 2 and 3) and two healthy volunteers (HD#5 and #7) were left untreated or treated with PMA/ionomycin for 30 or 60 minutes. Whole cell lysates were subjected to Western blotting using antibodies directed against the N-terminus of p105/p50 or against the phosphorylated form of p105. Anti-GAPDH and anti-β-actin antibodies were used as controls. Expression of p105 and p50 in cells carrying the heterozygous missense change p.Tyr350Cys only gains approximately half of the control levels. Whereas basal phospho-p105 is only detectable in healthy donor samples, a transient increase is observed in all panels. Expression analyses of p105 and p50 in EBV transformed B lymphocytes gained similar results and are shown in **Supplementary Figure 3**.

Our *in vitro* tests using transfected cells indicated that the p.Tyr350Cys missense change causes accelerated decay of the p105 precursor. Therefore, to test whether the c.1049A>G variant causes a heterozygous loss of expression *in vivo*, we analyzed the protein levels of p105, phospho-p105, and p50 in isolated PBMCs derived from two heterozygous mutation carriers (Patients 2 and 3) by Western blotting (**Figure 5**). In unstimulated cells from both patients, expression of p105 and p50 was markedly reduced to only half (or less) of the levels observed with the healthy donor controls. Similarly, upon stimulation with PMA/ionomycin, p105 and p50 levels in patient cells gained only approximately 50% of the intensities of the controls. In unstimulated cells, phospho-p105 was weakly detectable only in healthy donor samples. Upon stimulation, a transient increase of phosphorylated p105 (probably from cytoplasmic p105 pools) was observed in all samples analyzed, indicating that p105 (expressed from the non-mutant allele in patient-derived cells) remains unaffected and contributes to NF-κB signaling. However, the p50 levels in heterozygous cells remained at half of the dose observed in wildtype controls. Reduced p105/p50 levels were also confirmed in patient-derived EBV-immortalized B cells (**Supplementary Figure 3**). Since p105 levels are markedly reduced in patient-derived cells (probably due to accelerated mRNA and/or protein decay of the mutant form), mutant mature p50-Tyr350Cys proteins are probably not produced in relevant amounts (or are immediately eliminated once produced by processing of mutant p105 precursors). We therefore concluded that the disease-phenotype in FamA is most likely caused by the insufficient availability of both p105 and p50. Thus the heterozygous missense variant can elicit a p105/p50 (haplo)insufficient condition *in vivo*, reminiscent of previously reported severe truncating mutations.

### The Mutant p50-Tyr350Cys Interferes With Core Components of NF-κB1 Signaling

**Figure 6:**
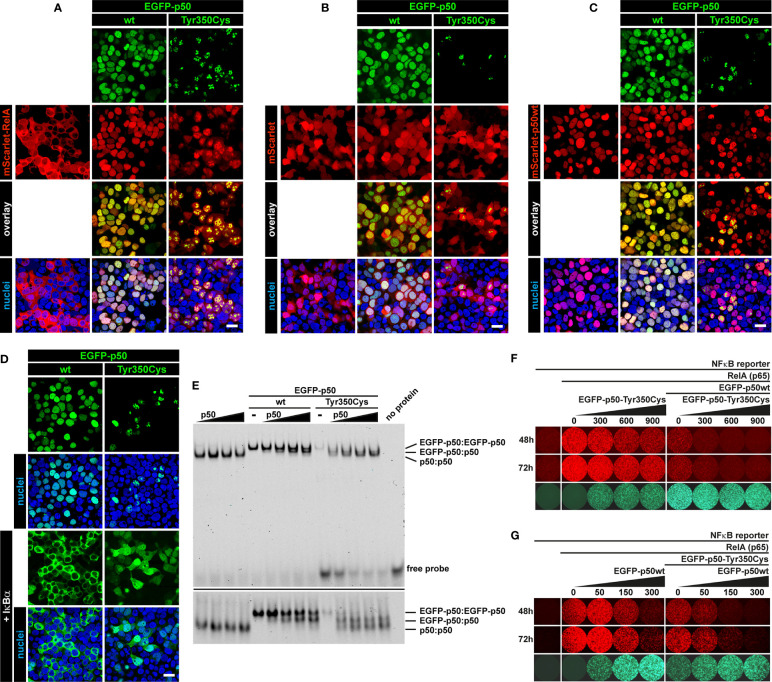
Interaction of mutant p50-Tyr350Cys with RelA, p50 and IκBα. HEK293T cells were transiently (co-)transfected with the indicated vector constructs. DNA amounts were titrated in pre-experiments for each panel to enhance the visibility of protein-protein interactions. Representative examples are shown. **(A)** Confocal imaging shows most of the ectopically expressed mScarlet-RelA(p65) fusion protein (red) localizing to the cytoplasm. Strong and/or prolonged overexpression of RelA had cytotoxic effects (data not shown). Upon co-expression of wildtype p50 or p50-Tyr350Cys (green), RelA shows a nuclear localization and adapts the homogeneous pattern of wildtype p50 or the aberrant subnuclear accumulation of p50-Tyr350Cys. Nuclei were stained with Hoechst 33342 (blue). Scale bar: 20µm. **(B)** The empty mScarlet-C1 vector was used as a control. The non-specific localization of mScarlet (red) is not influenced by the wildtype or mutant EGFP-p50 fusion proteins (green). **(C)** The majority of the nuclear EGFP-fused p50-Tyr350Cys (green) accumulates in high intense spot-like structures, whereas most of the co-expressed mScarlet-fused wildtype p50 (red) remains unaffected and shows its typical homogeneous localization. **(D)** Co-expressed non-fused IκBα (not visible), a cytoplasmic inhibitor of NF-κB1 dimers, leads to a virtually complete cytoplasmic retention of wildtype EGFP-p50 (green). The retained EGFP-p50-Tyr350Cys (green) apparently undergoes cytoplasmic decay (as indicated by attenuated fluorescence signals; not shown) whereas a proportion of the mutant protein localizes to the nucleus and adapts a uniform localization (at low abundance) or shows protein aggregation (at higher abundance). The fluorescence intensities were adjusted for better visibility. **(E)** Increasing amounts of non-fused wildtype p50 (lower molecular weight) were co-expressed with wildtype or mutant EGFP-fused p50 (higher molecular weight) and DNA-binding competition was determined by EMSA. DNA-binding of p50(wt):p50(wt) homodimers, EGFP-p50(wt or mutant):p50(wt) mixed homodimers and EGFP-p50(wt or mutant):EGFP-p50(wt or mutant) homodimers is indicated by low, medium and high band sizes, respectively. An identical gel was prepared with an approximately 2.5-fold running time to separate the DNA-protein bands (lower panel). An intermediate sized band indicates the interaction of wildtype and mutant p50 (right panel). Diverse binding partner preferences are obvious. **(F)** Cells were transfected with the NFκB-reporter vector alone (100 ng) as background control, plus RelA(p65) (5 ng) to switch on reporter expression (tdTomato, red), and plus EGFP-fused p50 (300 ng) (green) to demonstrate its inhibitory effect on RelA-mediated reporter activity. In the presence of p50 (and thus repressive p50:p50 homodimers) reporter activity is further repressed by increasing relative amounts of mutant p50-Tyr350Cys, whereas p50-Tyr350Cys alone has a dose-dependent attenuating effect. Numbers indicate transfected DNA amounts (ng) **(G)** Reverse promoter reporter experiment with increasing relative amounts of wildtype p50 alone or in the presence of co-transfected p50-Tyr350Cys (300 ng). The dose-dependent repressive effect of wildtype p50 is further intensified by the co-expressed p50-Tyr350Cys.

Our observations suggest insufficiency of both p105 and p50 due to intensified precursor decay (if the precursor is synthesized at all) as the primary molecular defect associated with the c.1049A>G/p.Tyr350Cys missense change. To test whether additional defects could potentially disturb NF-κB1-mediated signaling we analyzed whether the mutant p50-Tyr350Cys retained the capability to interact with RelA, wildtype p50, and IκBα (**Figure 6**). Confocal microscopy using co-transfected HEK293T cells demonstrated that mScarlet-fused RelA adapted the homogenous nuclear localization of EGFP-tagged wildtype p50, but also the aberrant subnuclear accumulation pattern of the EGFP-labelled mutant p50-Tyr350Cys (**Figure 6A**). No subcellular re-localization effect was seen, when the non-fused mScarlet alone was co-expressed together with wildtype or mutant p50 (**Figure 6B**). In contrast, co-expression of wildtype p50 together with its mutant counterpart led to a selective disposal of the p50-Tyr350Cys into subnuclear aggregates, while most of the wildtype p50 showed normal homogeneous nuclear localization (**Figure 6C**). Interestingly, the co-expressed IκBα caused an almost complete cytoplasmic retention of the EGFP-fused wildtype p50 (**Figure 6D**; left panel****), whereas the IκBα-mediated cytoplasmic re-localization of EGFP-p50-Tyr350Cys caused a reduction of EGFP signal intensities probably due to cytoplasmic protein decay (data not shown). A portion of p50-Tyr350Cys however escaped IκBα-dependent cytoplasmic retention and decay (possibly due to diminished interaction or “IκBα consumption”) and was still able to enter the nucleus (**Figure 6D**; right panel****) and to form aggregates. Using an EMSA-based DNA-binding competition assay we not only confirmed that the mutant EGFP-fused p50-Tyr350Cys interacts with its non-fused p50 wildtype counterpart but also showed that the mixed homodimers composed of a wildtype and a mutant p50 molecule can bind to DNA (**Figure 6E**). In addition, mutant p50-Tyr350Cys itself had an attenuating effect on RelA-mediated reporter activation, and further enhanced the repressive effect of wildtype p50, both in a dose-dependent manner (**Figure 6F**). Furthermore, the dose-dependent repressive effect of wildtype p50 was further intensified by its mutant counterpart (**Figure 6G**). Collectively these observations show that the mutant p50-Tyr350Cys retains the ability to interact with the three core-components of NF-κB1 signaling, RelA, p50 and IκBα. Therefore, additional functional effects cannot be excluded, although high expression levels of the mutant p50-Tyr350Cys and/or its precursor p105-Tyr350Cys are not sustained *in vivo*.

## Discussion

### The Pathogenic *NFKB1* c.1049A>G/p.Tyr350Cys Variant Causes Variable Clinical Phenotypes

In this study, we characterized a deleterious *NFKB1* missense variant in four members of a German family with a highly variable clinical presentation of CVID. Analyses of patients-derived cells and the use of a conventional *in vitro* transfection model consistently proved the pathogenicity of the sequence change. Unexpectedly, the single base pair/amino acid change caused a deleterious defect provoking a NF-κB1 (haplo)insufficient condition. Heterozygous pathogenic variants in *NFKB1* and *NFKB2*, the genes encoding the central components of the canonical and non-canonical NF-κB signaling pathways, have both been associated with primary antibody deficiencies (11, 12). Recently, employing systematic sequencing efforts in affected individuals, *NFKB1* mutations have been identified as the most frequent and second most frequent genetic cause of CVID in an European and an US cohort, respectively (22, 24, 38). For yet unknown reasons, pathogenic *NFKB1* variants are characteristically associated with a highly variable disease phenotype. In addition, it is not unusual that within the same affected family, some mutation carriers remain asymptomatic or have mild infections whereas others suffer from severe recurrent infections (20). In two large cohorts of patients with *NFKB1* variants, infections, autoimmunity, lymphoproliferation and malignancy were common clinical manifestations, with an age-dependent onset of respective symptoms (22, 24). Hypogammaglobulinemia and reduced switched memory B cells were the most frequently observed immunologic findings in symptomatic subjects. These phenotypic observations are confirmed in the present study: In the four patients, clinical symptoms varied significantly in particular with regard to onset and severity of infections. Patient 4 suffered from severe life-threatening bacterial infections since childhood, whereas patient 2 and 3 mostly suffered from recurrent rather mild viral upper airway infections. Patient 1 had no severe or frequently recurring infections until the age of 60. Autoimmunity and lymphoproliferation was observed in patient 1 (diabetes, arthritis, thyroid disease), patient 4 (ITP, splenomegaly) and 2 (mild vitiligo, splenomegaly). Due to the age-dependent onset of symptoms in patents with NF-κB insufficiency (24), we cannot exclude further manifestations later in life, particularly in the younger patients 2, 3 and 4.

Despite the variation in clinical presentations, our patients showed several consistent immunologic abnormalities, that have also been observed in *NFKB1* cohorts: All patients had different degrees of hypogammaglobulinemia (IgG and IgA). IgG prior to IgG substitution was lowest in the most severely affected patient 4. In the three patients tested, a normal IgG response to vaccine antigens was observed, consistent with a previous report, where reduced responses to vaccine antigens were found in only 65.2% of affected individuals (24). Reduced switched memory B cells are mostly observed in symptomatic *NFKB1* mutation carriers (13). All our patients had a reduction of switched memory B cells. Again, lowest numbers were seen in the most severely affected patient 4. Interestingly, all four patients had a decreased lymphocytic proliferative response to the B cell mitogen SAC, a finding that has to our knowledge not been reported previously. However, a correlation between the percentage of switched memory B cells and *in vitro* IgG synthesis after stimulation of B cells with SAC and IL-2 has been described (39). Thus, a reduced proliferative response upon stimulation with SAC may solely reflect low numbers of B cell subsets.

In patient 1, 2 and 3, IgG substitution was critically discussed due do non-severe infections, moderate hypogammaglobulinemia and regular specific antibody response upon vaccinations. Genetic testing and the presented results from functional studies however, supported the indication for long-term IgG substitution.

### Assessing the Pathogenic Potential of *NFKB1* Sequence Variants

The various molecular protein defects delineated from the huge number of *NFKB1* sequence variants identified to date can be assigned into at least four sub-groups (40). The first category includes severely truncating mutations and internal deletions typically affecting the N-terminal “p50 half” of p105. If not abolished by mRNA decay, these variants predict the expression of shortened, probably non- or dysfunctional proteins, or defective precursor proteins, respectively, which might undergo elimination and thus cause reduced levels of p105 and/or p50 as a prominent and recognizable feature. The second group comprises truncating variants affecting the central segment of p105. These predict the immediate expression of mutant p50-like proteins (assuming that protein synthesis can occur), thereby skipping the precursor stage, and retain the nuclear localization sequence. The majority of sequence alterations in *NFKB1* is represented by missense variants and can either affect the C-terminal half of the p105 precursor (and therefore are removed in case processing can occur) or the N-terminal p50 half (and therefore affect both, the precursor and the mature protein). Although the effect of single amino acid changes can be estimated using popular *in silico* analysis tools (again assuming each variant to be compatible with protein expression), experimental analyses are required to assess their pathogenic potential. However, convenient functional test using “indicators” of defective NF-κB signaling associated with immunodeficiency, such as altered expression of downstream targets or changes of surface markers, have not yet been established.

### Diagnostic Value of *NFKB1* Sequence Variant Testing Using *In Vitro* Models

Western blotting using isolated PBMCs has successfully been applied to confirm reduced levels of p105 and p50 associated with pathogenic truncating variants, precursor skipping variants and several missense variants (12–14, 22, 23, 34). EBV-transformed B lymphocytes might be used as an alternative (12, 14; **Supplementary Figure 3**), e.g. when sufficient blood samples are unavailable. However, it appears conceivable, that – in contrast to the p.Tyr350Cys variant - other single amino acid changes, although being pathogenic, might not cause devastating protein defects and thus might not cause overall reduced p105/p50 levels in primary cells. Rather, such variants might cause subtle molecular defects such as p105 precursor instability or delayed p50 nuclear translocation, which yet can be uncovered using *in vitro* modelling of the *NFKB1* sequence changes (15). Such approaches are commonly based on cloning of individual missense variants into a cDNA expression vector e.g. by site-directed mutagenesis, followed by ectopic expression of the modified p105 or p50 alongside with their wildtype counterparts in a heterologous cell culture system. A strong advantage is that the identification of protein-damaging variants can be accomplished solely with the knowledge of a given sequence change. Repeated blood sampling is usually not required, which might be of particular importance, when pediatric patients are to be diagnosed or where timely shipping is not warranted.

Biochemical analysis of the decaying protein itself in patient-derived (blood) cells might be challenging if not impossible (because the protein might be absent). In this regard, forced overexpression of the missense-mutant p50 and its subnuclear deposition is (only) an experimental maneuver to confirm its deleterious character and likewise represents a pure experimental artifact originating from its overabundance. An important additional concern when analyzing the protein localization in primary cells is the spheroidal cell shape. Indeed, we were unable to clearly discriminate between *NFKB1* wildtype and mutant cells using immunofluorescence staining of EBV-transformed B lymphocytes (data not shown). We therefore switched to a HEK293T overexpression model, yet any other transfectable cell line with a clear cytoplasm-nucleus structure such as fibroblasts or HeLa cells might be equally suitable.

In the current study, we used transient overexpression of (mostly) EGFP-fusion constructs in HEK293T cells, to test the subcellular localization by fluorescence microscopy, the “expressibility” and precursor processing by Western blot, the DNA-binding activity by EMSA and RelA-dependent target promoter activation in reporter assays. Protein interactions were tested by a combination of these assays. Our results show that the mutant precursor (or its processing product) undergoes abnormally high decay and thus predict, as an extrapolation, that a mutant p50-Tyr350Cys is not occurring *in vivo*. The immediate and forced expression of p50-Tyr350Cys (instead of being produced from its precursor) however, indicated additional molecular defects of the mature protein as evidenced by subnuclear mislocalization. These observations imply that missense changes assumed to affect the mature p50 – solely based on the amino acid position – need a careful assessment, since the major lesion might predominantly affect the precursor protein (40). The finding that a single amino acid change can cause a p105 and p50(haplo) insufficient condition was not expected and was not predictable. Yet, we do not expect our method to be potent enough to trace any conceivable p50 protein defect. Furthermore, mutations in the C-terminal half of p105 might cause precursor-specific defects. These might not be detectable, if sufficient wildtype p50 is generated by processing of the ectopically expressed mutant precursor (which removes the C-terminally located mutation), and if only the activity of the mature p50 is analyzed (e.g. DNA-binding).

Because our model is based on “overriding” the endogenous NF-κB, analysis of the transfected protein itself might be a valid attempt. Yet, conclusions should be carefully drawn from alterations of endogenous targets, differentially expressed genes, upstream or downstream NF-κB signaling processes or signaling outcome *per se*. In the present case, forced overexpression in a cell line model does certainly not reflect the p105 and p50 haploinsufficient condition observed in patient-derived cells (it is not even the contrary) and is therefore highly artificial. However, overexpression of p105 together with the experimental maneuver to short-cut the proteosomal processing step and to directly overexpress the mutant p50, makes a potential protein defect immediately “visible”. Particularly, the subnuclear accumulation, occurring when severely damaged p50 proteins are overexpressed, cannot be observed in patient-derived cells (data not shown), since the mutant precursor undergoes decay (if it is synthesized) and is not further processed. In general, overexpression models might not be sensitive enough to detect less devastating protein defects such as delayed signaling or alterations of binding partner preferences. Cell lines, with genetically engineered NF-κB components resembling strictly physiological conditions might be advantageous but have not been implemented yet.

### The Significance of the p105 Precursor

In the canonical NF-κB pathway, the mature p50 is generated by limited proteolysis of its p105 precursor, which can occur either by a co-translational/constitutive or by an inducible mechanism (41). In PBMCs (and other cells) both proteins are detectable under non-stimulated conditions by Western blotting. In addition, upon transfection, only a particular, apparently invariable proportion of p105 is converted to p50 by endogenous processes. The sustained presence of the precursor suggests that it executes additional autonomous functions - besides its “passive” role of being a mere resource for p50 processing. These include IκB-like functions mediated by the C-terminal protein domains. Therefore, control mechanisms that rapidly eliminate deleteriously damaged p105 proteins appear reasonable. Missense changes might lead to misfolding and thus trigger proteasome-mediated degradation.

The Tyr350Cys variant maps near the C-terminal end of the Rel-homology domain close to the nuclear localization sequence. However, the nuclear localization sequence is inactive in the cytoplasmic precursor and the amino acid change does not abolish nuclear entry of the mature p50-Tyr350Cys. Similarly, DNA-binding, which is also mediated by the Rel-homology domain, is not a task of the cytoplasmic precursor and apparently remains unaffected in the mature p50-Tyr350Cys. The interaction with dimerization partners (RelA and p50) and the binding to the cytoplasmic inhibitor IκBα - further functions of the Rel-homology domain – have also been addressed in the present study and likewise remained unaffected (or possibly was slightly impaired in the case of IκBα) in the mature p50-Tyr350Cys. Apparent differences such as the RelA-mediated reporter activity, which was decreased upon co-transfection of the p105-Tyr350Cys precursor but increased with the p50-Tyr350Cys, compared to the wildtype proteins, are plausibly explainable by the relative abundance of the proteins. Taken together, the molecular defect associated with the Tyr350Cys remained obscure. Besides misfolding of the p105-Tyr350Cys precursor, an attractive hypothesis might be the loss of the stop-signal for proteasomal processing. By proceeding towards the N-terminus of the precursor, complete proteolysis avoids the generation of a defective p50 (42). Alternatively, nonsense-mediated mRNA decay might protect the cells from producing dysfunctional p105/p50 proteins *in vivo*. In fact, we cannot exclude that the primary defect of the c.1049A>G variant affects mRNA stability. The p50 haploinsufficiency variant c.730+4A>G (causing the in-frame deletion of exon 8) however, allows expression of a mutant p105Δex8 precursor in patient-derived cells (12). When artificially overexpressed (to by-pass the precursor processing step), the nuclear p50-Tyr350Cys was disposed into aggregates, which can also include RelA, and p50-mediated transcriptional repression was enhanced upon co-expression of p50-Tyr350Cys. Thus, additional negative effects on NF-κB signaling – besides effects caused by insufficient p105 and/or p50 amounts - cannot be excluded.

Deciphering the abundance and the relative sub-cellular localization of RelA might provide an important key to understand the pathogenic consequences of insufficient p105/p50 protein amounts. Clearly, insufficient p105/p50 might disturb the balanced ratios of the NF-κB signaling components both, under steady-state conditions and during pathway stimulation and therefore perturb the NF-κB signaling dynamics. For instance, it will be interesting to see, whether less p105, and therefore less IκB-like activity mediated by its C-terminal half, *de facto* causes an increase of nuclear RelA. Alternatively, insufficient amounts of p50 could lead the preferential assembly of RelA homodimers or favor other dimer compositions due to stoichiometric imbalances and likewise reduce the transcriptional repressor activity otherwise mediated by p50 homodimers. In this regard and highly speculative, reduced p105/p50 would implicate an overall increased NF-κB activity. The availability of convenient experimental models in combination with reliable *in silico* prediction tools will be an important advancement for the assessment of the pathogenic potential of *NFKB1* missense variants. These models will help to illuminate the molecular mechanisms by which defective NF-κB1 signaling provokes defects of the immune system.

Our study underscores the importance of a thorough family history in PID, followed by immunologic and genetic testing, even of mildly affected family members, as well as functional assays to prove pathogenicity of identified variants. Although a genetic diagnosis with an increased risk for malignancy and autoimmunity may unsettle mildly affected or asymptomatic mutation carriers, the benefits of a close follow-up with early diagnosis and treatment of antibody deficiency, autoimmune or malignant manifestations may justify this approach.

## Data Availability Statement

The raw data supporting the conclusions of this article will be made available by the authors, without undue reservation.

## Ethics Statement

This study was carried out in accordance with the recommendations for studies with human subjects of the scientific committee at the University Medical Center of Freiburg. All physicians confirmed that their patients had signed an informed consent under local ethics-approved protocols and in accordance with the Declaration of Helsinki. The study protocol was approved by the ethics committee of the University Medical Center of Freiburg (Approval No. 295/13_200149 and 93/18_191111). No financial incentive was provided, neither to the patients nor the contributing physicians. Data was reported pseudo-anonymized, and physician-to-physician contact allowed to communicate treatment results and advice. Written informed consent was obtained from the individuals and the minor`s legal guardian for the publication of any potentially identifiable images or data included in this article.

## Author Contributions

MF designed and performed experiments prepared figures and wrote the experimental part of the manuscript. RK cared for patients, collected clinical and immunological information, summarized data and wrote the clinical part of the manuscript. SS performed Western blot experiments with patient-derived cells and analyzed data. LH cared for patients, provided clinical, immunological and experimental information. SB performed experiments and analyzed data. VW and HB cared for patients, provided clinical and immunological information and revised the manuscript. BG coordinated and supervised the study and revised the manuscript. All authors contributed to the article and approved the submitted version.

## Funding

This study was supported by the Deutsche Forschungsgemeinschaft (DFG, German Research Foundation) under Germany’s Excellence Strategy (CIBSS – EXC-2189 - Project ID 390939984 and RESIST - EXC-2155 - Project ID 39087428) and by the collaborative research center SFB1160/IMPATH to BG. BG received financial support from the E-rare programme of the EU, managed by the Deutsche Forschungsgemeinschaft (DFG), grant code GR1617/14-1/iPAD and from the “Netzwerke Seltener Erkrankungen” by the Bundesministerium für Bildung und Forschung (BMBF), grant code: GAIN_01GM1910A, and a collaborative research grant between Merck and UKL-FR (grant# ZVK2018073002). The article processing charge was funded by the Baden-Wuerttemberg Ministry of Science, Research and Art and the University of Freiburg in the funding programme Open Access Publishing.

## Conflict of Interest

The authors declare that the research was conducted in the absence of any commercial or financial relationships that could be construed as a potential conflict of interest.

## Abbreviations

ANK, Ankyrin-repeat domain; CMV, Cytomegalovirus; CVID, Common variable Immunodeficiency; DD, Death domain; DMEM, Dulbeccco´s Modified Eagle Medium; EDTA, Ethylenediaminetetraacetic acid; EGFP, Enhanced green fluorescent protein; ENT, Ear, nose and throat; EMSA, Electrophoretic mobility shift assay; FACS, Fluorescence activated cell sorting; FCS, Fetal calf serum; HRP, Horseradish peroxidase; IEI, inborn errors of immunity; IKK, IκB kinase; ITP, Idiopathic thrombocytopenia; IVIG, Intravenous immunoglobulin; NFKB1, Nuclear factor kappa B subunit 1; NF-κB, Nuclear factor ‘kappa-light-chain-enhancer’ of activated B cells; NIK, NF-κB inducing kinase; NLS, Nuclear localization sequence; NK cells, Natural killer cells; PBMC, Peripheral blood mononuclear cells; PHA, *Phytohemagglutinin;* PID, Primary immunodeficiency; PWM, Pokeweed mitogen; PVDF, Polyvinylidene fluoride; RHD, Rel-homology domain; RIPA, Radioimmunoprecipitation assay; RPMI, Rosewell Park Memorial Institute; SAC, *S. aureus* Cowan strain; SCIG, Subcutaneous immunoglobulin; SDS, Sodium-Dodecyl-Sulfate; VUS, Variant of uncertain significance; WES, Whole Exome Sequencing.
