# Supplementary material for: A Pathogenic Missense Variant in NFKB1 Causes Common Variable Immunodeficiency Due to Detrimental Protein Damage
Source: Front Immunol. 2021 Apr 27;12:621503. doi: 10.3389/fimmu.2021.621503 (PMC8115018; doi:10.3389/fimmu.2021.621503)
Supplement: Supplementary file 1 [file DataSheet_1.pdf]

Supplementary Material

Supplementary Figure 1

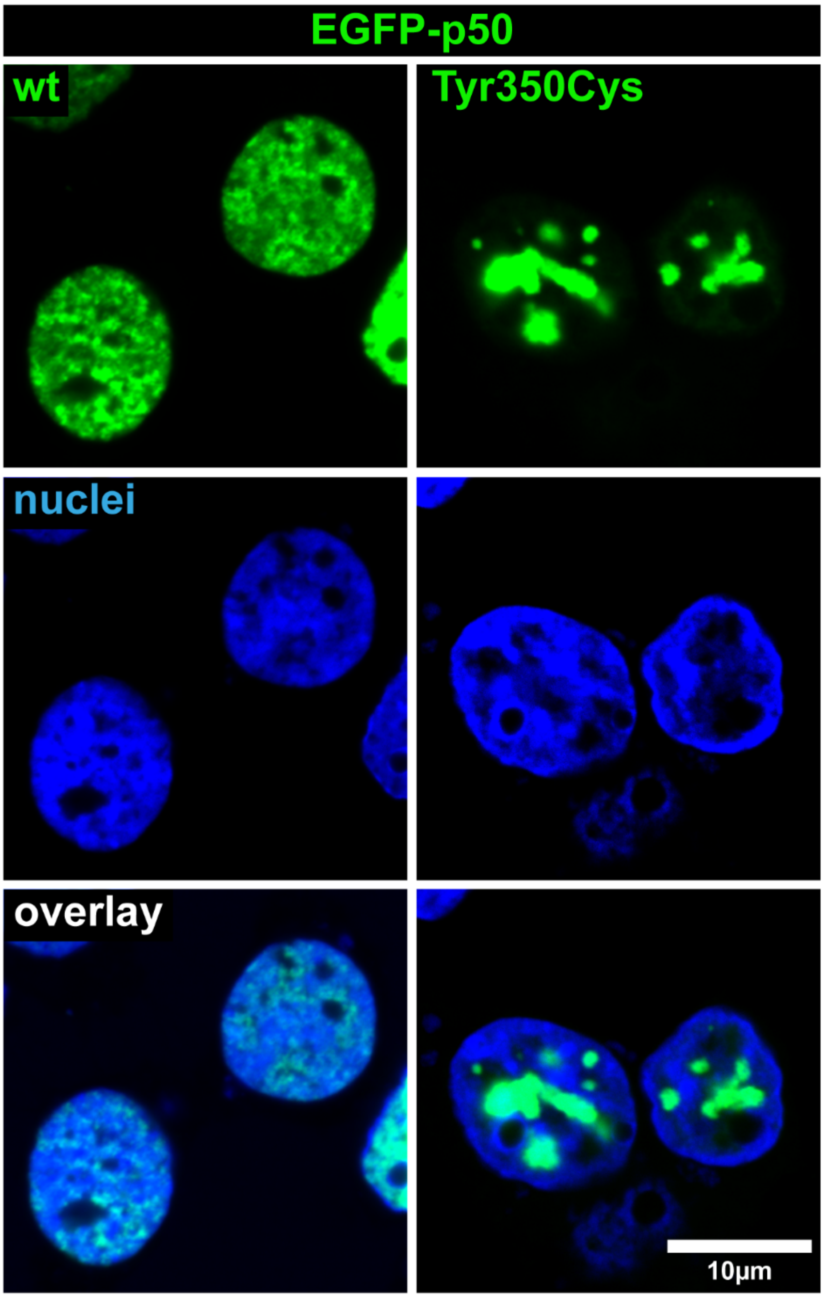

Supplementary Figure 1. The single amino acid change p.Tyr350Cys causes subnuclear mislocalization of p50.

Fluorescence imaging of EGFP-tagged proteins (green) confirms a uniform intra-nuclear localization of wildtype p50 whereas p50-Tyr350Cys accumulates in high-intense spot-like structures within the nuclei of transfected HEK293T cells. Higher magnification of an experiment as shown in Figure 2. Scale bar as indicated.

Supplementary Figure 2

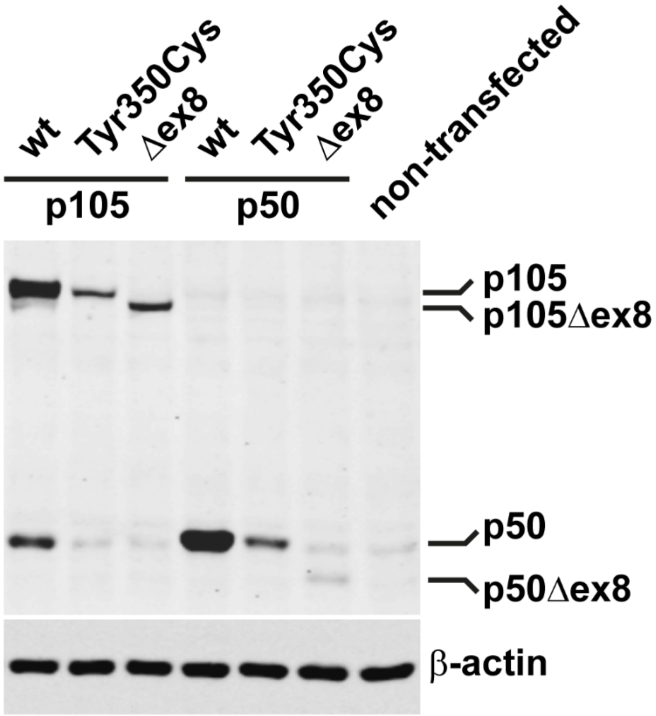

Supplementary Figure 2. Text. Limited expression levels of p105-Tyr350Cys and p50-Tyr350Cys in transiently transfected HEK293T cells.

Cells were transfected with expression vectors encoding non-fused versions of wildtype (wt) or mutant p105 or p50 as indicated (300 ng each per well on 48-well plates). The variant cDNAs carry either the Tyr350Cys missense change or the internal deletion Δex8 as a control. Whole cell lysates were prepared 40 hours after transfection and analyzed by Western blotting for expression and processing of p105 and expression of p50, respectively, using an antibody directed against the N-terminus of both p105 and p50. In transfected cells, ectopic wildtype p105 is sustained at robust expression levels. By cell autonomous mechanisms, a proportion of the ectopic wildtype p105 is processed to generate an excess of ectopic p50 compared to endogenous p50 levels (please see the non-transfected control). The mutant p105-Tyr350Cys gains only moderately expressed levels and processing to p50-Tyr350Cys is almost undetectable, resembling the deleterious internal deletion variant p105Δex8. In contrast to the ectopic wildtype p50, mutant p50-Tyr350Cys is not sustainable in transfected cells, again resembling the deleterious variant p50Δex8. Please note the marginal levels of the endogenous p105 and p50 proteins (faint bands in each lane), which supports the basic concept of “overriding” the endogenous NF-κB and of analyzing the transfected proteins in these cells. An anti-β-actin antibody was used to confirm equal loading. Representative results are shown. An analogous experiment using EGFP-tagged proteins is shown in Figure 3A.

Supplementary Figure 3

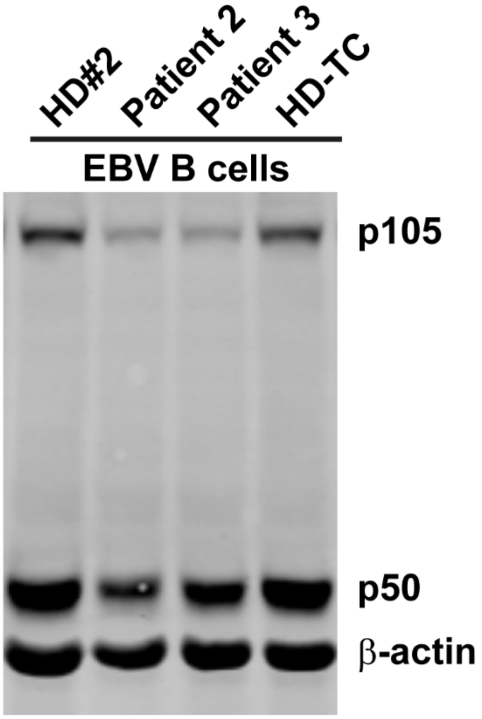

Supplementary Figure 3. Confirmation of p105 and p50 insufficiency using patient-derived EBV transformed B lymphocyte cells.

Immortalized B cell lines were generated by standard methods from PBMCs derived from two affected family members (Patients 2 and 3) and two healthy volunteers (HD#2 and HD-TC). Whole cell lysates were subjected to Western blotting, simultaneously using rabbit antibodies directed against the N-terminus of p105/p50 and mouse antibodies against β-actin as loading control. In cells with the heterozygous missense mutation c.1049A>G/p.Tyr350Cys expression levels of both p105 and p50 are reduced to approximately half of the levels observed in the healthy donor controls.
